# Loneliness and Mental Health Risks of Older People Living Alone in the Community: Cross-Sectional Study Protocol

**DOI:** 10.3390/healthcare14213725

**Published:** 2026-11-03

**Authors:** Odete Araújo, Cláudia Rodrigues, Fátima Braga, Rui Novais, Sandra Carreira, Maria do Perpétuo S. S. Nóbrega, Silvana Martins

**Affiliations:** 1 School of Nursing, University of Minho, 4710-057 Braga, Portugal; fbraga@ese.uminho.pt (F.B.); rnovais@ese.uminho.pt (R.N.); 2 Nursing Research Centre, University of Minho, 4710-057 Braga, Portugal; claudiailrodrigues15@gmail.com (C.R.); up201202120@up.pt (S.C.); 3 Health Sciences Research Unit: Nursing (UICISA: E), Nursing School of Coimbra (ESEnfC), University of Coimbra, 3045-043 Coimbra, Portugal; 4 Instituto de Ciências Biomédicas Abel Salazar (ICBAS), University of Porto, 4050-313 Porto, Portugal; 5 School of Nursing, University of São Paulo, São Paulo 05403-000, Brazil; perpetua.nobrega@usp.br; 6 ProChild CoLAB Against Poverty and Social Exclusion—Association, 4810-225 Guimarães, Portugal; silvana.martins@prochildcolab.pt

**Keywords:** mental health, aged, aging in place, loneliness, depression, frailty, social support, health literacy, cognitive function, falls

## Abstract

**Background/Objectives**: Some available evidence indicates an exponential rise regarding the proportion of older adults residing alone worldwide, which is associated with potential health and social risks, particularly those linked to loneliness and other mental health risk factors. This research aims to: (i) describe the physical and mental health profile of older people (aged over 65 years) living alone in the community, (ii) to examine the associations among the variables encompassing their mental health profile, and (iii) to explore the association between mental health and sociodemographic and health characteristics of this population. **Methods**: A cross-sectional study will be carried out. Potential participants must live alone in the selected municipality and be aged 65 years or older. Participants will be recruited through convenience sampling. Eleven instruments will be administered to participants assessing sociodemographic and health characteristics; fear of falling; sleep quality; frailty; anxiety, depression, and stress; loneliness; cognitive status; emotional literacy; perceived hope; functionality; and perceived social support. Data will be analysed using descriptive, univariate, and bivariate statistical methods. The study and its protocol were reviewed and approved by the Ethics Committee of a major public university in Northern Portugal (CEICVS 067/2022). **Expected Results**: This study aims to assess the physical and mental health of older adults living alone in a selected municipality in Northern Portugal, an area where such comprehensive data remain scarce. By conducting this analysis, we intend to inform and support the development of a highly personalized, evidence-based action plan, ultimately contributing to the mitigation of mental risk factors in this population.

## 1. Introduction

Population aging is a globally recognized phenomenon and has been widely studied across various academic disciplines, from the social sciences to the health sciences. Aging represents, first and foremost, one of the greatest civilizational achievements, reflected in the ability of individuals to live longer and in the best possible health. Secondly, and as a consequence of the expected health issues associated with biological aging, older individuals increasingly require support to mitigate the losses associated with the aging process [1]. The evolution of contemporary societies has led to profound sociodemographic changes among individuals and families, who increasingly have less ability to care for close relatives, whether it be due to geographical distance or professional obligations. As a result of these changes in the form and structure of families, more and more older people are living alone in their homes and communities.

Some of the available evidence suggests an exponential increase in the number of people living alone worldwide. For example, in the United States, more than 29% of 65 years old people live alone [2]. In Canada, the numbers are above of 19% [3], and we can observe a similar scenario in Japan (19.4%) [4]. According to Eurostat, over the last decade, the number of older people living alone has increased; in 2023, 20% of European older people lived alone [5].

There are potential health and social risks associated with isolation and loneliness. In particular, loneliness received increased attention during the COVID-19 pandemic, underscoring the need to further investigate and study older adults living alone and the potential risks associated with loneliness [6,7,8]. One of the most frequent mental health issues among older persons, though not exclusive to it, is anxiety and depression. Assuming that data on these conditions are often underestimated in many contexts, recent studies report a prevalence of 20% to 30% of older adults living alone experiencing anxiety and depression-related disorders [9,10]. The aging process is accompanied by losses that compromise the physical, cognitive, and social capacities of older adults. Consequently, the decline in these abilities increases their vulnerability to a range of risks. Falls, for example, are widely recognized as a public health issue, and the fear of falling (ptophobia) is particularly prevalent among older adults in general and especially among those who live alone [11,12]. The systematic review and meta-analysis conducted by Canever et al. [13] highlight the prevalence of sleep disorders in the elderly population, showing that, in addition to factors related to medication management, social isolation and loneliness also contribute to the worsening of sleep quality in older adults.

The main objectives of this study are: (i) to describe the physical and mental health profile of older people (aged over 65 years) living alone in the community, (ii) to examine the associations among the variables encompassing their mental health profile, and (iii) to explore the association between mental health and sociodemographic and health characteristics of this population.

## 2. Materials and Methods

### 2.1. Study Design

This is an exploratory and descriptive study and will be conducted using a cross-sectional design.

### 2.2. Participants and Setting

The study population will comprise community-dwelling older adults living alone in a specific municipality within the district of Braga, Northern Portugal.

Eligibility for participation will be determined according to the following inclusion criteria: (i) age ≥ 65 years; (ii) living alone; (iii) residency in the designated municipality. Individuals will be excluded if they (i) are unable to provide informed consent or (ii) reside in nursing homes or cohabit with relatives.

### 2.3. Sample

The study will be conducted with the support of local stakeholders, including the municipality, parish councils, and other relevant community services, which will assist in identifying potentially eligible participants. Based on the most recent available population data from the 2021 Census and municipal demographic records, the target population comprises approximately 507 community-dwelling older adults aged ≥65 years living alone in the selected municipality. Participants will be recruited using a convenience sampling technique and screened according to the predefined eligibility criteria. Based on an estimated target population of 507 individuals, a 95% confidence level, a 5% margin of error, and a 50% response distribution, the minimum required sample size was estimated at 219 participants. Therefore, the study will aim to recruit at least 219 eligible older adults. The municipality is not identified in the manuscript in order to preserve the confidentiality of the study setting during the peer-review process.

### 2.4. Outcome Measures and Instruments

In this study, community-dwelling older adults will first be evaluated using a questionnaire designed to gather information on both sociodemographic and health-related factors. Additionally, this instrument will enable a more comprehensive assessment of specific dimensions of social support, namely informal and formal care. A detailed operational definition of each variable is presented in Table 1.

Furthermore, the assessment of mental health risks will rely on a set of instruments that have undergone rigorous validation procedures and cultural adaptation for use within the Portuguese population.

These instruments capture a broad range of variables, including functionality [14]; frailty [15]; fear of falling [16]; cognitive status [17]; loneliness [18]; sleep quality [19]; anxiety, depression and stress [20]; emotional literacy [21]; perceived hope [22]; and perceived social support [23]. Further details regarding each instrument are summarized in Table 2.

### 2.5. Data Collection

The data collection will be conducted by researchers from the School of Nursing at the University of Minho in collaboration with scholarship holders recruited through a public selection process, which will be formally announced in a specific notice addressed to the academic community.

Data collectors will receive prior training and guidance on the administration of the questionnaires by a specialist in mental health and geriatric nursing in order to ensure procedural consistency and reduce potential bias.

The procedure will be carried out in accordance with the availability of the participants and based on prior agreement with the research team, established by telephone contact. The data will be collected in person at the civil parish council’s offices of the selected municipality. Moreover, older adults with mobility limitations will be brought to the civil parish council’s offices through coordinated transportation, using vehicles provided by the respective municipality. When transport is not feasible, home visits constitute an alternative strategy.

All instruments will be administered by trained interviewers in a face-to-face format rather than self-completed by participants. This approach was adopted to facilitate the participation of older adults with low educational attainment, literacy difficulties, visual impairment, or other functional limitations.

Data collection will be conducted in phases to accommodate participants’ health status and functional capacity. It is anticipated that participants will complete the full set of instruments in approximately 60 min. Participants will be offered breaks whenever necessary during the assessment. If fatigue, discomfort, or reduced concentration is observed, data collection may be temporarily interrupted and completed at a later time or during an additional session, according to participants’ preferences and availability. During this time, members of the research team will be available to assist participants in the completion of the questionnaires.

Illiterate participants will not be excluded from the study. Educational level, including illiteracy, will be recorded as a sociodemographic variable. The Portuguese version of the Montreal Cognitive Assessment (MoCA) will be administered by trained interviewers according to standardized procedures, and education-adjusted scoring recommendations will be applied during data analysis, as established in the Portuguese validation study.

Each instrument will be identified with a unique alphanumeric code to ensure participant anonymity, indicate the sequence of data collection, and identify the corresponding parish.

### 2.6. Data Analysis

Data will be analyzed using the Statistical Package for Social Science Software program (SPSS Inc., Chicago, IL, USA), version 30.0.

Descriptive statistics will be used to characterize the sociodemographic, physical health, functional, cognitive, psychosocial, and mental health profiles of community-dwelling older adults living alone. Categorical variables will be summarized using absolute and relative frequencies, where continuous variables will be described using means and standard deviation or medians and interquartile ranges, as appropriate.

To address the first study objective, descriptive analysis will be conducted for all study variables.

#### 2.6.1. Bivariate Analyses and Comparisons Between Groups

To address the second study objective, associations among the mental health profile indicators will be examined using Pearson’s or Spearman’s correlation coefficients, as appropriate to the measurement level, distributional characteristics, and assumptions of each analysis.

To address the third objective, differences in mental health outcomes according to sociodemographic and health characteristics will be examined using independent-samples *t*-tests for comparisons between two groups and one-way analysis of variance (ANOVA) for comparisons involving three or more groups, where appropriate. The choice of procedure will consider the research question, measurement level, sample size, group balance, and relevant statistical assumptions. Distributional characteristics, residuals, and homogeneity of variances will be assessed. Welch’s *t*-test or Welch’s ANOVA will be used when appropriate to account for unequal variances. When parametric procedures are not appropriate, alternative methods, such as the Mann–Whitney U test or Kruskal–Wallis test, will be considered in accordance with the research question and the properties of the data. These non-parametric procedures will not be treated as direct tests of mean differences.

When the research question specifically concerns differences between group means, bootstrap-based confidence intervals and inferential procedures may be considered as an alternative approach to estimating uncertainty, provided that the sample size, sampling design, independence of observations, and resampling procedure support their use. Bootstrapping will not be considered a universal remedy for violations of normality or other model assumptions. Where applicable, post hoc comparisons will account for the variance structure and multiplicity of testing. Effect sizes and confidence intervals will be reported alongside significance tests.

Multivariate analysis of variance (MANOVA) may be considered to examine group differences across multiple correlated mental health outcomes, particularly the depression, anxiety, and stress subscale scores of the Depression Anxiety Stress Scales (DASS-21), provided that the sample size, research question, and relevant multivariate assumptions support this approach. If these conditions are not met, outcome-specific analyses will be conducted as appropriate.

#### 2.6.2. Multivariable Regression Analyses

Multivariable regression models will be specified according to the study objectives and a priori conceptual framework. The principal outcomes for regression analyses will be the DASS-21 depression, anxiety, and stress subscale scores and the global score of the Pittsburgh Sleep Quality Index (PSQI). Separate models will be fitted for each outcome, subject to sample size and the suitability of the data for the proposed model.

For the second objective, will explore the associations between each principal mental health outcome and other relevant indicators of the mental health profile, including loneliness, perceived social support, perceived hope, and emotional literacy. The selection of explanatory variables for each model will be guided by the conceptual framework and the specific research question. Other DASS-21 subscale scores will not be included automatically as explanatory variables in models where another DASS-21 subscale is the outcome; their inclusion will require a clear theoretical justification and assessment of multicollinearity.

For the third objective, separate multiple linear regression models will be fitted for depressive symptoms, anxiety, stress, and sleep quality. Candidate explanatory variables will include age, sex, education level, frailty, cognitive status, functionality, and fear of falling. Loneliness, perceived social support, perceived hope, and emotional literacy may also be included when justified by the study objectives and conceptual framework. The final covariate set will be specified before model fitting, taking into account the available sample size, conceptual relevance, potential confounding, and risk of overfitting. Continuous variables, including age and scale scores, will generally be retained in their continuous form rather than categorized arbitrarily.

For linear regression models, results will be reported as regression coefficients (B), 95% confidence intervals, and *p*-values. Model diagnostics will assess linearity, residual distribution, homoscedasticity, multicollinearity, and influential observations. If the assumptions or data characteristics do not support the proposed model, an alternative analytical approach will be considered and justified according to the outcome and research question.

#### 2.6.3. Interaction Terms

Interaction effects will be examined only where supported by a clear theoretical rationale and an adequate sample size. One potential exploratory interaction is between loneliness and perceived social support in the model for depressive symptoms, to investigate whether the association between loneliness and depressive symptoms varies according to perceived social support. If examined, the model will include both main effects and the interaction term. Continuous predictors will be mean-centred before calculating the interaction term to facilitate interpretation. The interaction coefficient and its 95% confidence interval will be reported, and conditional effects or adjusted predictions will be examined where appropriate. This analysis will be identified as exploratory.

#### 2.6.4. Missing Data and Data Quality

Data quality will be assessed by checking completeness, coding consistency, plausibility of values, duplicate records, and potential data-entry errors. The extent and pattern of missing data will be examined for each variable, including their relationship with observed participant characteristics where feasible. The number of observations included in each analysis will be reported.

The extent and pattern of missing data will be examined for each variable, including their relationships with observed participant characteristics where feasible. The number of observations included in each analysis will be reported. Complete-case analysis will be considered when missing data are negligible and there is no substantial indication that excluding incomplete observations would introduce bias. When missing data warrant imputation, multiple imputation using regression-based methods available in IBM SPSS Statistics will be considered, provided that the missingness assumptions, sample size, variable characteristics, and available information support this approach. Relevant analysis variables and suitable auxiliary predictors will be considered in the imputation models. Estimates from the imputed datasets will be pooled using Rubin’s rules. Where feasible, sensitivity analyses comparing imputed and complete-case results will be conducted. If imputation is not methodologically appropriate, the extent and potential implications of missing data will be reported transparently.

The significance level adopted for the study’s analysis tests will be 0.05 [24]. Given the number of outcomes and comparison, results will be interpreted in conjunction with effect sizes, confidence intervals, and the potential for multiplicity.

### 2.7. Ethical Considerations

This study and its research protocol were approved by the Ethics Committee of a major public university in Northern Portugal (CEICVS 067/2022).

All eligible older people will receive comprehensive and detailed information regarding the aim, purpose, and variables included in the present study. Furthermore, participants will be explicitly informed that their participation is entirely voluntary and that they may drop out at any point without any adverse consequence.

In addition, each participant will be required to provide written informed consent, in person, to ensure compliance with all ethical principles inherent to the conduct of the research, particularly the safeguarding of participants’ identity and the confidentiality of the information collected.

## 3. Discussion

This protocol proposes a structured assessment of the physical and mental health of older adults residing alone in a municipality in Northern Portugal, a region for which comparable data are still lacking. The findings of the present study are expected to support the development of a highly personalized, evidence-based action plan, ultimately contributing to mitigating mental risk factors in this population.

The literature indicates an increased risk of adverse health outcomes associated with loneliness [6,7]. Loneliness and social isolation have become topics of growing research interest, particularly in the post-pandemic context, driven by evidence of their influence on both physical and mental health [8]. However, as highlighted by a recent integrative literature review, although older adults interact with healthcare professionals, the screening for loneliness remains uncommon, and few professionals actively assess its negative consequences among their patients [25].

The pioneering nature of this protocol lies in its adoption of a multidimensional approach that extends beyond the screening of loneliness to include the assessment of additional mental risk factors such as depression, anxiety, stress, fear of falling, cognition, and perceived social support. By employing instruments widely recognized by the scientific community, this study will explore the relationships between psychosocial and physical health variables, thereby contributing to a comprehensive understanding of the prevalence and severity of these factors among community-dwelling older adults.

Based on previous studies, a significant proportion of participants is expected to report high levels of loneliness, despite all of them living alone [25]. Furthermore, it is plausible that levels of loneliness will vary across parishes, with higher values anticipated in rural areas, as geographical location may influence opportunities for interaction and social connection [7].

In the study by Moeyersons et al. [26], community-dwelling older adults with reduced mobility perceived loneliness as an inherent aspect of aging, arising from the cumulative losses, limitations, and perceived lack of meaning in this stage of life. Frailty in older adults has been conceptualized as a multidimensional concept comprising multiple losses in physical, cognitive, and social domains [27]. Recent evidence demonstrates that loneliness and social isolation may contribute to an increased risk of frailty [28]. Furthermore, a significant association between loneliness and both the presence and progression of frailty among community-dwelling older adults has been found [29]. However, the mechanisms underlying this association remain unclear. The present study seeks to address this gap by examining the relationship between the distinct dimensions of frailty and loneliness within this population.

Loneliness and social isolation have also been widely associated with cognitive decline in older adults, although they appear to influence cognitive function through distinct mechanisms [30]. Recent longitudinal evidence [31] suggests that loneliness is a significant predictor of lower cognitive functioning among people aged 65 years and older, particularly accelerating decline in memory and verbal fluency. According to the authors, the impact of loneliness tends to intensify with advancing age, possibly due to the increasingly critical role that social interactions play in maintaining cognitive function. These findings support the hypothesis that loneliness constitutes a modifiable psychosocial risk factor, underscoring the importance of preserving social relationships [31].

Anxiety, depression, and stress are highly prevalent among older adults. Depression has reached alarming levels, with estimated prevalence rates ranging from 19.2% [9] to 31.74% [10]. In addition, older adults aged 50 years and older have demonstrated that loneliness was significantly associated with an increased risk of depression, even among those who were not socially isolated or who reported having social support. This association persisted over a 12-year follow-up, indicating that loneliness, regardless of other social factors, has a sustained impact on the mental health of this population [32]. These findings are supported by a Spanish study which found that the protective effects of education and financial status diminished when loneliness was considered, further highlighting its central role in the risk of depression among older adults [33]. In the present study, a high prevalence of depression is anticipated, along with a significant association between loneliness and depression. These expectations underscore the need for interventions focused on reducing social isolation, particularly by enhancing more meaningful relationships and connections [32].

Several studies have highlighted the critical role of social support in mitigating depression among community-dwelling older adults. Wang et al. [34] provided substantial evidence indicating that a lower perception of social support is associated with poorer outcomes in terms of depression symptomatology, recovery, and overall functioning. Loneliness also emerges as a prominent risk factor, linking it to more symptoms of both depression and anxiety [34]. Complementing these findings, a meta-analysis [35] demonstrated that social support can be effective in alleviating depression within this population. In this context, the present study aims to deepen the understanding of the complex interrelationships between perceived social support, loneliness, and depression among community-dwelling older adults. In addition, it seeks to inform the development of a targeted community intervention and to reinforce the vital role of nurses in promoting mental health within this population.

Fear of falling is also a common concern among community-dwelling older adults [11,12]. A systematic review and meta-analysis [12] identified twenty-eight potential risk factors for fear of falling, highlighting anxiety and depression as the most significant psychological contributors. Similarly, MacKay et al. [11] reported a consistent association between depressive symptoms and fear of falling, alongside advanced age, female sex, previous falls, and poorer physical performance. Thus, Das Gupta et al. [36] found that adults aged 80 and above are more likely to report symptoms of depression, while those aged 60 to 69 with a diagnosed depressive disorder are more disposed to experience falls. Considering these findings, the present study aims to advance current knowledge by examining how physical and psychological factors, particularly anxiety and depression, are associated with fear of falling among community-dwelling older adults, while considering disparities across distinct sociodemographic profiles.

Fear of falling is also a risk factor and predictor of functional limitations in older people. In a longitudinal study conducted with community-dwelling older people, those who lived alone and had a history of falls and/or fear of falling had fewer mobility limitations in the following year [37]. In light of these findings, exploring the level of functionality of community-dwelling older people is particularly relevant, and it is expected that they do not present high levels of functional limitation.

Recent evidence found that a proportion of older adults also experience sleep problems or have suboptimal sleep health [38]. Thus, it seems that community-dwelling older adults who feel lonely and socially isolated are more likely to experience insufficient sleep, which suggests that loneliness, social isolation, and health concerns may be related to sleep quality [39,40,41]. In our study, we also acknowledge the likelihood that a portion of participants may exhibit poor sleep quality. Building on previous evidence, we also anticipate a relationship between loneliness and sleep quality among our study participants.

Previous studies have found that psychological variables such as hope can significantly influence the well-being of older adults. In particular, a stronger sense of hope is positively associated with better physical health, as evidenced, for instance, by fewer sleep problems [42]. Moreover, higher levels of hope appear to correlate with reduced psychological distress and greater psychological and social well-being, including increased life purpose and satisfaction.

In addition to the variables analyzed, health literacy represents a particularly concerning issue among older adults. Research consistently indicates that low levels of health literacy are prevalent in this population and are associated with poorer physical, mental, and social health outcomes. A systematic review [43] identified multiple factors contributing to inadequate health literacy in older adults, namely sociodemographic, social, economic, and health-related factors. Considering the characteristics of our study population, it is likely that several of these risk factors will be present. Advanced age, lower education levels, limited social support, loneliness, frailty, cognitive decline, and mental health problems are expected, all of which suggest a higher likelihood of low health literacy levels.

Emotional literacy, as a core element of health literacy, has garnered increasing attention in recent years. A recent study [44] showed that positive emotional expressivity is negatively associated with social loneliness. Their findings indicate that older people are, to a certain extent, capable of mitigating social loneliness by expressing their true positive emotions. This suggests that promoting the expression of positive emotions, while reducing the negative, may serve as an effective strategy for preventing social loneliness [44]. Through our study, we aim to contribute further evidence to support these findings in community-dwelling older adults.

### Limitations and Future Implications

Despite its relevance to a still underexplored area within the national context, this study may present several methodological limitations commonly associated with research involving older adults.

A primary challenge concerns the identification and recruitment of participants. In the Portuguese cultural setting, living alone in old age remains relatively uncommon, despite demographic trends indicating a growing proportion of older individuals residing alone. This living arrangement is often associated with increased vulnerability and social stigma, which may hinder participant engagement—particularly regarding comprehension of the study’s aims and willingness to participate. These barriers may require extended data collection efforts and may result in a smaller sample size, thereby limiting the generalisability of the findings.

Furthermore, participants will be recruited through a convenience sampling strategy, which may limit the representativeness of the sample and lead to selection bias. It should be noted that these findings may not be entirely generalizable to all community-dwelling older adults living alone in Portugal or in other geographical settings and should therefore be interpreted with caution.

Secondly, given the cross-sectional nature of this study, causal inferences cannot be established, and findings should be interpreted as associations observed at a single point in time. Despite its limitations, this study can provide relevant contributions by describing the physical and mental health profile of older adults living alone in a district of the Northern region. Based on the expected evidence, local stakeholders will be able to develop action plans focused on promoting successful aging, mitigating health and social risks that could lead to hospitalization, institutionalization, or premature death. Additionally, this study is expected to support the design and implementation of structured programs, specifically targeting mental health promotion among older adults living alone.

## Figures and Tables

**Table 1:** Sociodemographic questionnaire: operational definition of variables.

Sociodemographic and Health Variables	Definition
Gender	Female, Male, Other
Age	Years
Education level	I cannot read or write, I can read and write, but I did not attend school, I attended school but did not complete the 1st cycle of primary school, First cycle of primary school, Second cycle of primary school, Third cycle of primary school, Upper-secondary education, Vocational education and training, Bachelor’s degree, Master’s degree, Doctorate,
Marital Status	Single, Married, Divorced, Widowed
Time living alone	<1 year, 1–5 years, 6–10 years, ≥10 years
Informal sources of support	Sister/brother, Daughter/son, Granddaughter/grandson, Daughter-in-law/son-in-law, Friends, Neighbors, Others, Not applicable
Rate of informal support	Daily, 2 to 3 times per week, weekly, biweekly, monthly, or other
Mode of informal support received	In-person, by phone/mobile, by video call, not applicable, or other
Satisfaction with informal support received	Not satisfied, somewhat satisfied, satisfied, very satisfied, or not applicable
Formal care providers	Nurse, doctor, physiotherapist, psychologist, home care services, not applicable, or other
Frequency of formal care	Daily, 2 to 3 times per week, weekly, biweekly, monthly, or other
Perceived satisfaction with formal care	Not satisfied, somewhat satisfied, satisfied, very satisfied, or not applicable

**Table 2:** Overview of instruments used to assess variables associated with mental health risks.

Instrument	Measure	Study Authors	Number of Items	Dimensions	Cultural Adaptation and Validation	Rating Scale	Global Score Classification
Portuguese version of the Barthel Index for Activities of Daily Living	Functionality	Araújo etal. [14]	10 Bathing, dressing, feeding, grooming, bowels, bladder, toilet use, transfers, mobility, stairs	Mobility and self-care Bowel and Bladder Management	α = 0.96	Each item is scored in increments based on the level of independence: 0, 5, 10, or 15. 0—total dependency 15—independence	0–24: Total dependency 21–60: Severe dependency 61–90: Moderate dependency 91–99: Slight dependency 100: Full independence
Portuguese version of the Tilburg Frailty Indicator	Frailty	Coelho et al. [15]	25 Part A: 10 items Part B: 15 items	Determinants of frailty Total frailty: physical, psychological, and social	KR-20 = 0.78	All rated from 0 to 1 Dichotomous scale: 11 items Yes/No Trichotomous scale: 4 items No/Sometimes/Yes	Cut-off point for frailty: 6
European Portuguese version of the Falls Efficacy Scale– International	Fear of falling	Figueiredo & Santos [16]	16	Fear of falling	α = 0.978	4-point Likert scale: 1–4 ‘Not all concerned’ to ‘Very concerned’	Maximum: 64 Greater fear of falling
Portuguese version of the Montreal Cognitive Assessment	Cognitive status	Freitas et al. [17]	11	Attention, concentration, memory, working memory, executive function, temporal and spatial orientation, language, visuoconstructional skills	α = 0.94	1 point for each correct response	Maximum: 30 Better cognitive status
Portuguese version of the UCLA- Loneliness Scale	Loneliness	Pocinho et al. [18]	16	Social isolation Affinities	α = 0.905	4-point Likert scale: 1–4 ‘Never’ to ‘Always’	Cut-off point: >32 Negative feelings of loneliness
Portuguese version of the Pittsburgh Sleep Quality Index	Sleep quality	Del Rio João et al. [19]	24	Subjective sleep quality, sleep latency, sleep duration, habitual sleep efficiency, use of sleeping medication, daytime dysfunction	α = 0.70	4-point scale: 0–3	Cut-off point: >5 Major difficulties in ≥2 dimensions or moderate difficulties in >3 dimensions
Portuguese version of the Depression Anxiety Stress Scales	Anxiety, depression and stress	Pais-Ribeiro et al. [20]	21 3 subscales, each with 7 items	Anxiety ^1^ Depression ^2^ Stress ^3^	α = 0.74 ^1^; 0.85 ^2^; 0.81 ^3^	4-point Likert scale: 0–3 ‘Did not apply to me at all’ to ‘Applied to most of the time’	Maximum: 21 (each subscale) The most negative affective states
Portuguese version of the Emotional Literacy Skills Scale	Emotional literacy	Sousa et al. [21]	31	Motivation, empathy, self-regulation, emotional awareness, social skills	α = 0.755	5-point Likert scale: 1–5 ‘Never true’ to ‘Almost always true’	3.41–5.00: strong emotional literacy 2.61–3.40: need for attention and promotion of emotional literacy <2.60: priority intervention in emotional literacy
Portuguese version of the Perceived Hope Scale	Perceived hope	Marujo et al. [22]	6	Perceived hope	α = 0.88–0.89	6-point Likert scale: 0–5 ‘Strongly disagree’ to ‘Strongly agree’	Maximum: 30 High level of perceived hope
Multidimensional Scale of Perceived Social Support	Perceived social support	Carvalho et al. [23]	12 4 items for each subscale	Family Friends Significant other	α = 0.87–0.95	7-point Likert scale: 1–7 ‘Very strongly disagree’ to ‘Very strongly agree’	Maximum: 7 Greater social support is perceived

Note. A: Cronbach’s alpha, KR-20: Kuder-Richardson Formula 20. Superscripts 1, 2 and 3 are used to distinguish between the different alpha values.

## Data Availability

No new data were created or analyzed in this study.
